# Development of Novel Polymorphic EST-SSR Markers in Bailinggu (*Pleurotus tuoliensis*) for Crossbreeding

**DOI:** 10.3390/genes8110325

**Published:** 2017-11-17

**Authors:** Yueting Dai, Wenying Su, Chentao Yang, Bing Song, Yu Li, Yongping Fu

**Affiliations:** 1 Engineering Research Center of Chinese Ministry of Education for Edible and Medicinal Fungi, Jilin Agricultural University, Changchun 130118, China; daiyueting18@jlau.edu.cn (Y.D.); suwenying1001@jlau.edu.cn (W.S.); song19800123@jlau.edu.cn (B.S.); 2 China National GeneBank, Environmental Genomics, Beijing Genomics Institute, Shenzhen 518083, China; yangchentao@genomics.cn

**Keywords:** *Pleurotus tuoliensis*, expressed sequence tag-simple sequence repeat, monokaryon, genetic diversity, transferability

## Abstract

Identification of monokaryons and their mating types and discrimination of hybrid offspring are key steps for the crossbreeding of *Pleurotus tuoliensis* (Bailinggu). However, conventional crossbreeding methods are troublesome and time consuming. Using RNA-seq technology, we developed new expressed sequence tag-simple sequence repeat (EST-SSR) markers for Bailinggu to easily and rapidly identify monokaryons and their mating types, genetic diversity and hybrid offspring. We identified 1110 potential EST-based SSR loci from a newly-sequenced Bailinggu transcriptome and then randomly selected 100 EST-SSRs for further validation. Results showed that 39, 43 and 34 novel EST-SSR markers successfully identified monokaryons from their parent dikaryons, differentiated two different mating types and discriminated F_1_ and F_2_ hybrid offspring, respectively. Furthermore, a total of 86 alleles were detected in 37 monokaryons using 18 highly informative EST-SSRs. The observed number of alleles per locus ranged from three to seven. Cluster analysis revealed that these monokaryons have a relatively high level of genetic diversity. Transfer rates of the EST-SSRs in the monokaryons of closely-related species *Pleurotus* *eryngii* var. *ferulae* and *Pleurotus ostreatus* were 72% and 64%, respectively. Therefore, our study provides new SSR markers and an efficient method to enhance the crossbreeding of Bailinggu and closely-related species.

## 1. Introduction

*Pleurotus tuoliensis* [1], also known as Bailinggu, is one of the many significant, choice edible mushrooms [2]. Bailinggu was first cultivated successfully in China in 1986 [3]. In recent years, more research has been devoted to its genetic diversity, breeding and cultivation [1,2,3,4,5]. Presently, however, only six varieties are used for commercial cultivation. Therefore, strengthening the genetics and breeding of Bailinggu is urgently needed. 

Crossbreeding plays an important role in mushroom breeding [6,7], but it is seldom done with Bailinggu. Conventional procedures of mushroom crossbreeding include monokaryon isolation and identification from dikaryotic strains, determination of their mating types, cross-mating two monokaryotic mycelia, microscopic observation of clamp connections at the contact line and hybrid offspring detection [2,6,7,8]. However, these conventional methods are troublesome and time consuming. Simpler, more efficient and precise methods are urgently needed. One such method is molecular-assisted selection, which would benefit the application of mushroom genetics and breeding strategies.

Nowadays, molecular markers are key tools that support and expedite genetics and breeding programs [9,10,11]. Simple sequence repeat (SSR) markers are becoming more effective markers, largely due to their codominance, easy amplification, high reproducibility and multi-allelic variation [12]. Transcriptome sequencing is an efficient, cost-effective way to develop expressed sequence tag-simple sequence repeat (EST-SSR) markers. EST-SSR markers have been useful for the assessment of genetic diversity surveys, construction of genetic maps, cultivar identification and other related studies [5,13,14,15,16]. However, research related to the use of molecular markers for identifying monokaryons and their corresponding genetic diversity, mating types and hybrid offspring in Bailinggu breeding is limited [5,16]. 

In this study, we developed EST-SSR markers from a newly-sequenced transcriptome of Bailinggu using RNA-seq technology. The specific objectives of this study were to: (1) identify SSR loci in the transcriptome of Bailinggu and analyze their distribution, frequency and size; (2) develop novel EST-SSR markers for Bailinggu that can be used to differentiate monokaryons and their parent dikaryons, identify monokaryon mating types and genetic diversity and detect crossings of F_1_ and F_2_ hybrids with monokaryons; and (3) test the transferability of the novel EST-SSR markers in closely-related species *Pleurotus eryngii* var. *ferulae* and *Pleurotus ostreatus*. The newly-identified EST-SSR markers could become a valuable resource for further crossbreeding of Bailinggu and closely-related species. 

## 2. Materials and Methods

### 2.1. Isolation of Protoplast-Derived Monokaryons and Determination of Mating Types

Five dikaryotic strains of Bailinggu (CCMJ814, 973, 980, 1077 and 1123) were used to isolate protoplast-derived monokaryons (Table 1). The growth medium for the dikaryotic mycelia was a liquid MYG medium (10 g maltose, 5 g yeast extract powder, 5 g glucose and 1000 mL water). Incubation using static cultures was performed at 24 °C for seven to 11 days. Mycelia were then harvested by filtration, washed with 0.6 M D-mannitol (Dingguo, Beijing, China) as an osmotic stabilizer and incubated for 150 min at 27 °C in 2.0% lywallzyme (Guangdong Institute of Microbiology, Guangzhou, China) [9,17]. The obtained protoplasts were filtrated using cotton with 0.6 M D-mannitol and then resuspended in 6 M D-mannitol. The final concentration was approximately 1 × 10^6^ protoplasts/mL. Next, we cultured the diluted protoplasts on regeneration media (liquid maltose yeast glucose (MYG) medium added to 15 g agar and 0.6 M D-mannitol per liter) at 24 °C. The regenerated colonies from the protoplasts were examined under a microscope with fluorescent staining using 4’,6-diamidino-2-phenylindole (DAPI, Sigma-Aldrich, St. Louis, MO, USA) to observe clamp connections and the number of nuclei [18]. The monokaryon should only have one nucleus without clamp connections. 

To determine the mating types of monokaryons from the same parent strain, the identified monokaryons were subjected to a monokaryon-monokaryon (mono-mono) mating test at 24 °C on a solid maltose yeast glucose medium for two weeks using somatic incompatibility tests [3,9]. Mating mycelia at the contact zone were selected to observe the presence of clamp connections using a microscope. The presence of clamp connections indicates that the mating types of two monokaryons are different, while the absence of clamp connections indicates that the mating types of two monokaryons are the same. 

### 2.2. Isolation of Basidiospore-Derived Monokaryons

Seven dikaryotic strains of Bailinggu, including CCMJ967, 968, 973 (CCMJ973 was used to obtain protoplast- and basidiospore-derived monokaryons at the same time), 974, 1001, 1002 and 1044, were used to isolate monokaryons from basidiospore suspensions, performed as previously described by Larraya et al. [19]. The obtained monokaryons were also examined under a microscope with fluorescent staining with DAPI to observe clamp connections and the number of nuclei. Then, the identified monokaryons were subjected to a mono-mono mating test [2]. For the across-taxa transfer of the novel EST-SSR markers in the related species, *P. eryngii* var. *ferulae* and *P. ostreatus*, the basidiospore-derived monokaryons were derived from dikaryotic strains of CCMJ970 (*P. eryngii* var. *ferulae*) and CCMJ1080 (*P. ostreatus*) (Table 2).

### 2.3. Crossbreeding of Monokaryons

In order to obtain Bailinggu hybrid dikaryotic strains, we used the monokaryons obtained from 11 dikaryotic strains for intra-strain crossing. We then examined the presence of clamp connections in the obtained hybrid dikaryotic mycelia under a microscope. Among them, 72 hybridized combinations were successfully cross-mated and the hybrid F_1_ mycelia obtained. We then selected a hybridized combination of M28 × M33 to obtain F_1_ fruiting bodies and F_2_ population. Monokaryon M28 was obtained from the dikaryotic strain CCMJ1123 (D11), and monokaryon M33 was obtained from the dikaryotic strain CCMJ1077 (D2). To test fruiting of the F_1_ hybrid dikaryotic mycelia from M28 × M33, the mycelia were transferred to a new MYG plate and incubated at 24 °C for two weeks [2]. We next used plastic bottles as containers for the cultivation substrate, which contained 780 g of medium, including 35% sawdust, 5% corncob, 24% wheat bran, 10% maize powder and 4.5% soybean meal. These bottles were incubated at 24 °C for 60 days. Bottles were then processed by scraping off a part of the mycelia surface and moving them to a cropping room at 13–15 °C, with fluorescent lighting of 600–800 lx for 10 h and humidity of 93–95%. We harvested 13 F_1_ fruiting bodies within 30–50 days in the cropping room and gathered their spores. Single-spore isolation from F_1_ fruiting bodies and hybridization were performed to acquire the F_2_ population. All strains were maintained in the Engineering Research Center of the Chinese Ministry of Education for Edible and Medicinal Fungi of the Jilin Agriculture University, China.

### 2.4. Development of Novel Expressed Sequence Tag-Simple Sequence Repeat Markers Using Transcriptome Sequencing

The dikaryotic mycelia samples of Bailinggu (CCMJ1044) used for transcriptome analysis were kindly provided by the Hengdaxing mushroom factory (Beijing, China). The materials were handled with the same method described by Fu et al. [16]. To perform transcriptome analysis, the mycelia from four treatment stages were used including the control sample that was grown at 25 °C and three cold stage samples that were grown at −3 °C for 2, 6 and 10 d. All samples were immediately frozen in liquid nitrogen and stored at −80 °C. We used a TRIzol reagent (Life technologies, Grand Island, NY, USA) to extract the total RNA. We further evaluated the samples to ensure their eligibility by examining their purity, concentration and integrity with a Nanodrop 2000 UV-Vis spectrophotometer (Thermo Scientific, Waltham, MA, USA), a Qubit 3.0 Fluorometer (Life Technologies, Grand Island, NY, USA) and an Agilent Technologies 2100 Bioanalyzer (Santa Clara, CA, USA), respectively. We constructed 12 cDNA libraries (each stage had three replicates) and performed paired-end sequencing reactions on an Illumina HiSeq 2500 platform at Biomarker Technologies Co., Ltd. (Beijing, China). Sequencing data are available in the National Center for Biotechnology Information (NCBI) Sequence Read Archive (SRA), under Accession Number SRP090421. Raw reads were trimmed with adapters, and low-quality reads were filtered. We then used Trinity (http://trinityrnaseq.sf.net) [16] software to assemble clean, short reads from the 12 libraries, yield transcripts and further cluster into unigenes. Filtration was done after assembly, and the parameter set was relaxed slightly compared with our previous analysis [16] in order to obtain more useful data. 

MIcroSAtellite (MISA) (http://pgrc.ipk-gatersleben.de/misa/) [16] was applied to search for SSRs in 31,139 assembled unigenes. The criteria for the default parameters of the SSR loci were as follows: mono-, di-, tri-, tetra-, penta- and hexa-nucleotide motifs with a minimum of ten, six, five, five, five and five repeats, respectively. The maximum number of bases that interrupted two SSRs in a compound locus was 100. Because of unigene redundancy, we filtered SSRs by integrating those that were located in the same gene, but corresponded to different isoforms. Based on the above procedures, we investigated the characteristics of SSRs in the transcriptome of Bailinggu, including density (number of loci per Kbp), motif (type, length and repeat times) and location (coding sequence, non-coding sequence or untranslated regions).

### 2.5. Validation of Novel Expressed Sequence Tag-Simple Sequence Repeat Markers

Primer 3 software (http://www.premierbiosoft.com) [16] was used to design primer pairs for EST-SSRs, with the following criteria: polymerase chain reaction (PCR) product size was 100–400 bp, primer length was 18–22 nucleotides, GC content was 40–70% and annealing temperature was 60 °C. These markers were synthesized by Sangon Biotech Co., Ltd. (Shanghai, China). We then randomly chose 100 primer pairs to test whether they could successfully amplify with eight Bailinggu monokaryons (four from columnar-shaped strains and four from palmate-shaped strains).

The EST-SSR primer pairs that successfully obtained stabilized bands were used to differentiate monokaryons from their dikaryotic strains, identify monokaryon mating type, and distinguish F_1_ and F_2_ hybrids. Next, we investigated the genetic diversity of 37 Bailinggu monokaryons. First, the genomic DNA from fresh mycelia was extracted with the Plant DNA Mini Kit (Kangwei, Beijing, China), following the manufacturer’s instructions. Second, the PCR were carried out in 25-μL final reaction volumes, containing 20 ng template DNA, 1× buffer and 0.2 mM dNTPs, 0.2 μM primer pairs and 0.5 U Taq DNA polymerase (Dingguo, Beijing, China). PCR was performed using a Thermal Cycler (Bio-Rad, Brockport, NY, USA), with the following thermal cycling protocol: 5 min at 94 °C, then the samples were processed through 30 cycles for 30 s at 94 °C, 30 s at 60 °C and 30 s at 72 °C. All amplifications were completed with a 10-min extension step at 72 °C. The PCR products were separated by 8% (*w*/*v*) non-denaturing polyacrylamide gel electrophoresis in 1× tris-borate-EDTA (TBE) buffer. The polymorphic bands were visualized by silver staining [20]. Amplification products were scored as present (1) or absent (0). We used GenAIEx6.502 software [21] and NTSYSpc2.1 software (East Setauket, NY, USA) to calculate the genetic diversity parameters and construct an unweighted pair group method with arithmetic mean (UPGMA) dendrogram, respectively [22]. 

### 2.6. Across-Taxa Transferability of Novel Expressed Sequence Tag-Simple Sequence Repeat Markers in Monokaryons of Related Species of the Genus Pleurotus

Two closely-related species of the genus *Pleurotus* (*P. eryngii* var. *ferulae* and *P. ostreatus*) were selected to measure the across-taxa transfer rate of the new EST-SSR markers in their six single-spore-derived monokaryons. All 100 EST-SSR markers mentioned above were used to measure the transfer rate, which was calculated as the proportion of SSR primers that showed successful amplification in each species.

## 3. Results and Discussion

### 3.1. Isolation of Protoplast-Derived Monokaryons

We optimized the protocol for obtaining the monokaryons from Bailinggu by using the protoplast-derived method. We found that culturing liquid mycelia for 11 days was more effective than the traditional seven days. The rate of the protoplast-derived monokaryon development from regenerated colonies was more than 93% using 11-day-old liquid mycelia, compared to about 60% with a seven-day-old liquid. Microscopic analysis showed that a total of 162 protoplast-derived monokaryons were obtained from five dikaryotic strains of Bailinggu. Among them, 36, 4, 50, 50 and 22 protoplast-derived monokaryons were obtained from dikaryotic strains CCMJ973, 980, 1077, 1123 and 814, respectively. We used these monokaryons for further EST–SSR marker validation, genetic diversity estimation and Bailinggu hybridization. 

Monokaryons obtained from protoplasts were vegetative progeny taken directly from the nutrient hypha without undergoing meiosis. Therefore, we usually could obtain two different mating types (AxBx and AyBy) of protoplast-derived monokaryons. However, we found that there is an imbalance in the recovery of the two mating types from a dikaryon after protoplasting in this study. The mono-mono mating-type tests revealed that the four dikaryotic strains CCMJ973, 980, 1077 and 1123 produced monokaryotic strains that had the two mating types, while the dikaryotic strain CCMJ814 produced monokaryotic strains with only one of the mating types. Previous fungi studies also demonstrated that one or two mating type monokaryotic strains could be obtained from their parent dikaryotic strains through protoplast regeneration technology [9,23]. In addition, the ratio of two different mating types of monokaryons from the four dikaryotic strains CCMJ973, 980, 1077 and 1123 were 3:1, 3:1, 1.63:1 and 2.57:1, respectively. Theoretically, if protoplast-derived monokaryons are regenerated from the same dikaryon, the two mating types will be equally distributed [23]. However, in our study, the ratio of two different mating types ranged from 1.63:1–3:1. Lin and Zhang also demonstrated that the proportion of the two mating types is not always 1:1 in many types of fungi [24]. We infer that our imbalanced ratios might be caused by different protoplast regeneration rates and different mycelia growth rates of the diverse parent dikaryons. Therefore, we urgently need useful molecular markers to improve the identification efficiency of monokaryons and their mating types due to the imbalance that occurred in protoplast-derived monokaryons.

### 3.2. Novel EST-SSR Markers Developed Using Transcriptome Sequencing

Most research on the genetics and molecular biology of Bailinggu has concentrated on genetic diversity analyses of dikaryotic germplasm resources [1,5,16]. Consequently, effective molecular markers for use in monokaryon identification are limited, which in turn hinders crossbreeding of Bailinggu. Therefore, in order to broaden the range of molecular tools for Bailinggu, we developed a large number of EST-SSR loci using Bailinggu transcriptome data. With the Illumina HiSeq 2500, we employed a paired-end sequencing strategy on samples of Bailinggu mycelia. This technology has a high throughput and is an efficient, rapid and cost-effective tool for SSR mining, particularly for less well-studied species [25,26,27]. In this study, we obtained substantially new sequence data and SSR loci information for Bailinggu. We found that 2211 (5.66%) out of 31,139 unigenes harbored SSR loci and that 165 unigenes have more than one SSR locus. In total, 1110 EST-SSR loci were identified in these unigenes (Table 3). The density of EST-SSR loci in the transcriptome was 0.018 loci per Kbp, which was lower than *Grifola frondosa* (0.034 per Kbp) [28], *Agaricus subrufescens* (0.024 per Kbp) [27] and *Auricularia polytricha* (0.089 per Kbp) [29]. The discrepancy between SSR frequencies may be partially related to differences in species, SSR screening criteria or the amount of detected sequences.

The number of SSR loci identified in this Bailinggu study was approximately twice that of the previous report [16] because we relaxed the filtration parameters slightly after assembly with Trinity. In order not to affect our downstream analysis, the identified SSR loci were then filtered—those located in the same gene, but corresponding to different isoforms were integrated—because some unigenes were redundant. As a result, we increased the possibility for developing effective markers that can be used for further studies. 

**Figure 1:**
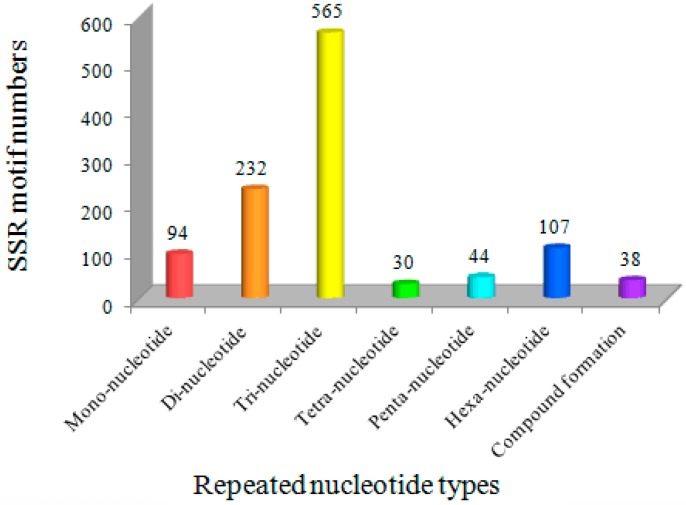
The distribution of SSR motif types in Bailinggu transcriptome.

The trinucleotide repeats (TNR) were the most common type of SSR motif (565, 50.90%), followed by dinucleotide repeats (DNR) (232, 20.90%), hexanucleotide repeats (HNR) (107, 9.64%), mononucleotide repeats (MNR) (94, 8.49%), pentanucleotide repeats (PNR) (44, 3.96%) and tetranucleotide repeats (TTNR) (30, 2.70%). In addition, 38 EST-SSRs were present in compound formations (Figure 1). The finding of TNR as the most abundant repeat motif in the Bailinggu transcriptome is similar to studies of many other fungi, such as *A. polytricha* [29], *Lentinula edodes* [30], *Boletus edulis* [31], *Volvariella volvacea* [32] and *Phellinus linteus* [14]. Therefore, a high frequency of tri-nucleotide repeats might be a common feature in EST-derived SSRs. Previous studies suggested that the TNR type of microsatellites should be generated from DNA replication slippage and would be less likely to cause frame-shift mutations in coding regions, resulting in a relatively lower impact on gene expressions [33]. However, an aberrance in other motifs types (except HNR) can lead to a variation of open reading frames, which can cause a frameshift mutation and lead to the formation of shorter or completely different proteins [34]. Meanwhile, the high frequency of DNR may be explained by their abundance in several codons with different nucleotide arrangements. Some DNR, such as (AG)n/(CT)n, are not selectively neutral and may have functional roles [35].

We identified more than 234 motif sequence types (including complementary sequences). The G/C motif appeared 58 times in MNR and was more prevalent than the A/T motif. Five tandem repeats were the most common repeat number (260, 46.02%) in TNR, followed by six tandem repeats in DNR (126, 54.31%) and TNR (107, 18.94%). In addition, the mean length of all 1110 SSRs was 21.76 bp, and the mean lengths of the different repeat types, in increasing order, were DNR (15.34 bp), MNR (16.48 bp), TNR (21.14 bp), TTNR (26.40 bp), PNR (23.98 bp) and HNR (27.70 bp). The largest number of repetitions was 56 for DNR and 39, 26, 21, 16 and 9 for MNR, TNR, TTNR, PNR and HNR, respectively. 

We next investigated the distributional characteristics of SSR loci in the unigenes from the Bailinggu transcriptome. Of these 1110 EST-SSRs, 131 and 490 were located in the coding sequence regions (CDS) and untranslated regions (UTR) respectively, and 192 were located over CDS such that each SSR locus was located in both the CDS and UTR regions. Due to the lack of enough information to delimit the CDS region in some unigenes harboring SSR loci, 297 EST-SSRs were not ascertained for their distributional characteristics. The density of SSRs in the UTR was three-times more than the density in the CDS. A similar pattern was also observed in previous transcriptome studies [35,36]. For SSR present in the CDS, TNR (63, 48.10%) was the most abundant followed by DNR (33, 25.19%), MDR (10, 7.63%), HDR (8, 6.11%), PDR (7, 5.343%) and TTDR (6, 4.58%). A high frequency of these SSRs located in transcribed regions, especially in the untranslated portions, could be attributed to mutation and selection pressure of amino acids [33,37]. For breeding, because of the association with important economic and agronomic traits, SSR loci located in the transcribed regions may be of more interest.

### 3.3. Validation of Novel Expressed Sequence Tag-Simple Sequence Repeats

**Figure 2:**
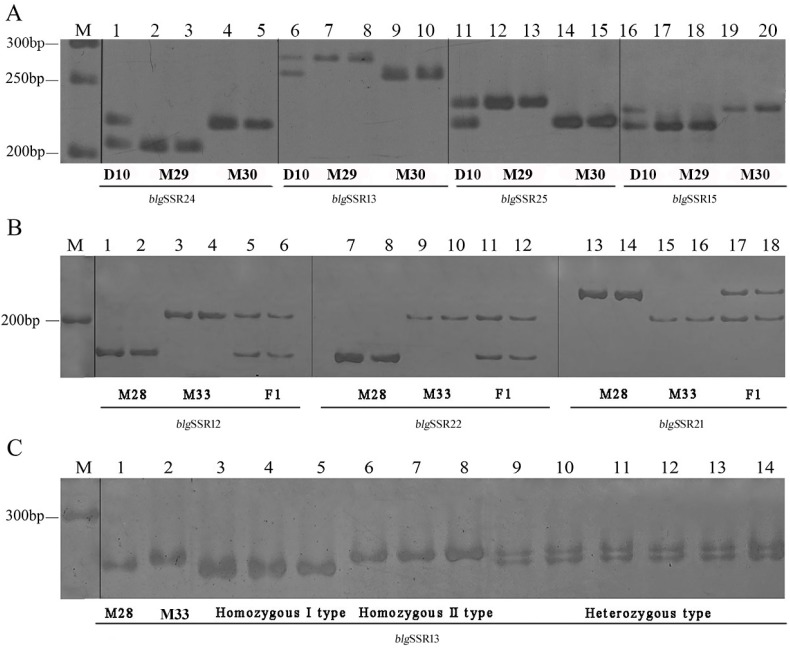
Examples of *expressed sequence tag-simple sequence repeat* markers applied in Bailinggu. (**A**) Polymorphic bands between monokaryons and their parent dikaryons using *blg*SSR24, *blg*SSR13, *blg*SSR25 and *blg*SSR15 (1, 6, 11, 16: parent dikaryons CCMJ973 (D10); 2–3, 7–8, 12–13, 17–18: monokaryon M29; 4–5, 9–10, 14–15, 19–20: monokaryon M30; the dikaryons CCMJ973 (D10) had two bands, while monokaryons M29 and M30 only had one band at the corresponding position); (**B**) polymorphic bands between the monokaryotic strains and their F_1_ hybrid using *blg*SSR12, *blg*SSR22 and *blg*SSR21 (1–2, 7–8 and 13–14: monokaryon M28; 3–4, 9–10 and 15–16: monokaryon M33; 5–6, 11–12 and 17–18: F_1_ hybrid; monokaryon M28 and monokaryon M33 only had one band, while their F_1_ hybrid was the combination of M28 and M33); (**C**) the monokaryotic strains and their F_2_ hybrid using *blg*SSR13 (1: monokaryon M28; 2: monokaryon M33; 3–5: F_2_ homozygous-like monokaryon M28; 6–8: F_2_ homozygous-like monokaryon M33; 9–14: F_2_ heterozygous).

We successfully designed 317 primer pairs using Primer 3. To identify the efficiency of these primer pairs and to screen for high-quality EST-SSR markers useful for crossbreeding of Bailinggu, we selected a set of 100 primer pairs (Supplementary Materials, Table S1). These primer pairs included 34 DNR, 54 TNR, 1 TTNR, 1 PNR, 3 HNR and 7 multiple repeats. Among these primers, 86 pairs (86.00%) showed a clear amplification in Bailinggu monokaryons, whereas the remaining 14 failed to amplify. These 86 EST-SSR markers will be useful in constructing a genetic linkage map for Bailinggu. We also developed 39 EST-SSRs (*blg*SSR1–6, 10–42) and 43 EST-SSRs (*blg*SSR1–38, 43–47) and used them to quickly and efficiently differentiate monokaryons from dikaryons and identify the mating types of monokaryons, respectively (Figure 2A).

Next, due to the codominance of SSR markers, we found 34 EST-SSRs (*blg*SSR9–19, 24–27, 36–41, 45–57) useful for identifying F_1_ and F_2_ hybrid offspring (Figure 2B,C). We also found that all individuals of the F_1_ generation had the same genotype and similar phenotype, which corresponds with Mendel’s law of uniformity. Moreover, the genetic separation ratio of the F_2_ generation using EST-SSR markers equaled 1:2:1 (homozygous like parent A: heterozygous: homozygous like parent B), which matches the F_2_ generation of plants [38]. Therefore, these newly-developed EST-SSR markers are effective and can be used for further genetic and breeding studies of Bailinggu. 

Conventional methods for crossbreeding of Bailinggu are troublesome and time-consuming, involving monokaryon isolation, antagonistic activity tests, mono-mono mating tests, microscopic observation and isozyme electrophoresis [39,40,41]. Additionally, to identify hybrids, the traditional detection method of esterase isozyme electrophoresis is quite complicated and is influenced by external conditions [42]. Therefore, we need to explore a new appraisal method that is simpler, more efficient and precise. The development of molecular markers is an efficient method to enhance crossbreeding of Bailinggu. Previous reports have shown that SSR markers are simple, highly stable, more efficient and more precise in the identification and discrimination of breeding materials [10,43]. For our study, using traditional methods to validate the 162 monokaryons and hybrids would have required more than 20–30 days. In contrast, using our newly-developed SSR markers for DNA preparation, PCR amplification and polyacrylamide gel electrophoresis detection, this same validation can be completed within 1–2 days. Compared with the traditional method of microscopic examination and isozyme electrophoresis for identifying Bailinggu’s hybrid offspring, SSR markers are faster and more accurate. SSR markers may also be applied to track related genotypes within the offspring. Above all, our newly-developed SSR markers will benefit and facilitate breeding research of Bailinggu.

### 3.4. Genetic Diversity Analysis of Monokaryons and Dikaryons Using 18 EST-SSRs

To analyze the genetic diversity of Bailinggu monokaryons, we selected 28 single spores derived monokaryons from seven dikaryotic strains and nine protoplast-derived monokaryons from five dikaryotic strains (Supplementary Materials Table S2) using 18 polymorphic, clear and stable EST-SSRs. The genetic parameters of these SSR markers are shown in Table 4. In total, 86 alleles were detected across all 37 monokaryons. The percentage of polymorphic loci was 100%. The observed number of alleles per locus (Na) ranged from three (*blg*SSR30) to seven (*blg*SSR24), with a mean value of 4.778 per locus. Shannon’s information index (I) value ranged from 0.616–1.587, with a mean of 1.138. The polymorphism information content (PIC) of the 18 EST-SSRs ranged from 0.321–0.766, with a mean of 0.584, which was higher than previously reported means for *Cordyceps militaris* (0.191) [44], *Flammulina velutipes* (0.420) [45] and *Auricularia auricula*-*judae* (0.470) [12], but lower than *Agaricus bisporus* (0.618) [46]. The PIC value was greater than 0.500, depicting a high discrimination power. Among these 18 EST-SSRs, 14 (*blg*SSR11, 13, 14, 16, 24, 26, 27, 28, 29, 32, 37, 38, 43 and 56) were high polymorphic loci with PIC values of more than 0.500; none were low polymorphic loci with PIC values less than 0.250. The higher PIC values for our EST-SSRs indicate that these polymorphic EST-SSR markers, derived from the mycelia transcriptome, have suitable power for discriminating among the monokaryons of Bailinggu. Thus, our EST-SSR markers will help broaden the gene pool and improve Bailinggu crossbreeding programs aimed at reducing costs and conserving energy. The maximum and the minimum genetic distance between the monokaryons was 1.022 and 0.000, respectively, with a mean of 0.641. These results indicated that the 37 monokaryons of Bailinggu had a relatively high genetic diversity. In further breeding programs, these polymorphic and stable EST-SSR markers can be used for construction of a genetic linkage map and quantitative trait locus (QTL) mapping for Bailinggu.

**Figure 3:**
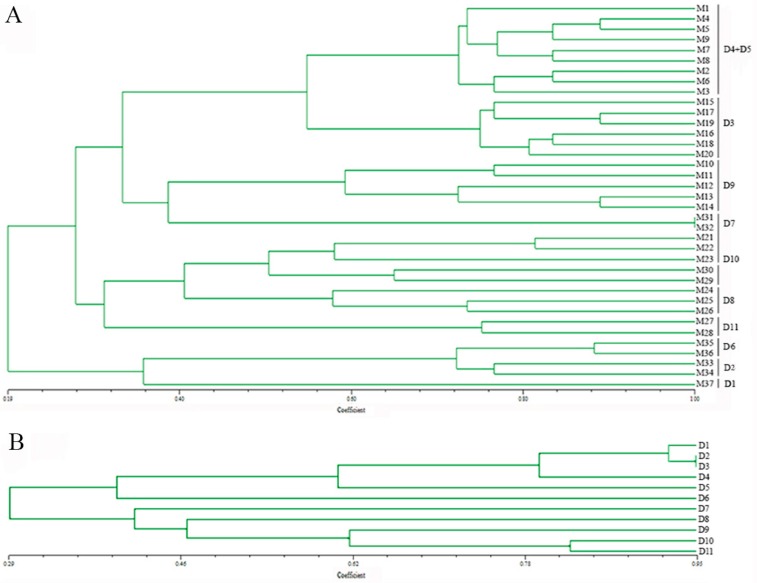
Unweighted pair group method with arithmetic mean (UPGMA) dendrograms of Bailinggu based on 18 novel EST-SSR markers. (**A**) UPGMA dendrogram of 37 monokaryons of Bailinggu based on 18 novel EST-SSR markers. M1–M37 represented monokaryons obtained from parent dikaryons. D1–11 represented the parent dikaryotic strains: D1-CCMJ814, D2-CCMJ1077, D3-CCMJ967, D4-CCMJ974, D5-CCMJ968, D6-CCMJ1044, D7-CCMJ980, D8-CCMJ1001, D9-CCMJ1002, D10-CCMJ973, D11-CCMJ1123. (**B**) UPGMA dendrogram of 11 parent dikaryotic strains of Bailinggu based on the same 18 novel EST-SSR markers.

According to the cluster analysis, based on the Dice similarity coefficient and UPGMA algorithm, the genetic similarity coefficient among all 37 monokaryons ranged from 0.19–1.00 (Figure 3A). Monokaryons derived from one dikaryotic strain were clustered into the same group, except monokaryons (M1–M9) derived from the dikaryotic strains CCMJ974 (D4) and CCMJ968 (D5). We discovered that these two wild dikaryotic strains were collected from Yumin, China, in 2012 and inferred that they may have the same genetic background. Combined analysis of monokaryons and dikaryons allows the possibility of more information about the markers, so we also analyzed the genetic relationship of the dikaryotic strains using the same 18 EST-SSR markers. We found that the genetic similarity coefficient between the 11 dikaryotic strains ranged from 0.29–0.95 (Figure 3B). This result was similar to the range of the clustered monokaryons. However, these dikaryotic strains have more heterozygosity associated with each locus. 

The above results showed that these Bailinggu strains from natural populations have a relatively high genetic diversity. We hypothesized that the natural genetic recombination may also contribute to the relatively high genetic diversity of Bailinggu populations. Therefore, we used these 18 EST-SSR markers to assess the possibility of natural genetic recombination occurring in these 11 dikaryotic strains of Bailinggu. Based on the PCR amplification products and non-denaturing PAGE results (Figure S1), four out of 18 EST-SSR markers (*blg*SSR 14, 30, 31, 56) could identified as having the natural genetic recombination in some of the 11 dikaryotic strains of Bailinggu, although they were from natural populations. These results indicated that not only does natural gene mutation contribute to the relatively high genetic diversity of Bailinggu populations, but also natural genetic recombination. 

### 3.5. Across-Taxa Transferability of Novel Expressed Sequence Tag-Simple Sequence Repeat Markers in the Monokaryons of Two Species of the Genus Pleurotus

To evaluate the possibility of the EST-SSR markers from the Bailinggu transcriptome used in monokaryons from closely-related species of the genus *Pleurotus*, we carried out across-taxa transferability analysis for these 100 novel EST-SSR markers in the monokaryons from *P. eryngii* var. *ferulae* and *P. ostreatus*. In the monokaryons of *P. eryngii* var. *ferulae*, 72 primers (*blg*SSR 2–14, 18, 20–23, 26–28, 31, 33, 34, 36, 38, 40–44, 46, 48–49, 52–54, 56–58, 60–82, 88–90, 93–98) were amplified products. In monokaryons of *P. ostreatus,* 64 primers (*blg*SSR 4–13, 15, 20–22, 25–27, 34, 36–37, 40–46, 48–54, 57, 59–82, 88, 95–98) were amplified products. Therefore, the overall transferability rates of these 100 novel EST-SSRs were 72% in *P. eryngii* var. *ferulae* and 64% in *P. ostreatus*. 

Previous studies showed the significant or moderate conservation of EST-SSR markers in the related species within the same genus in fungi and plants [47]. We also found relatively high transferability rates (82%) of EST-SSR markers developed from Bailinggu in the dikaryons of *P. eryngii* var. *ferulae* [5]. In addition, the across-taxa transferability rate in monokaryons of *P. eryngii* var. *ferulae* (72%) was higher than in *P. ostreatus* (64%). A likely reason for this difference is that *P. eryngii* var. *ferulae* has a closer genetic relationship with Bailinggu than *P. ostreatus*, resulting in moderate cross-species conservation of SSR-containing genes [1,5]. In fact, we found some unigenes known to encode conserved proteins in fungi contained in our SSR loci, such as unigenes that code hydrophobin (*blg*SSR31), ATP-dependent helicase (*blg*SSR10), PH_Scd1 and vWFA domain-containing protein (blgSSR15), the bZIP domain of ATF-2 and similar proteins (*blg*SSR52), the catalytic domain of cell division control protein 7-like serine/threonine kinases (*blg*SSR43) and some hypothetical proteins (*blg*SSR53, 63, 84). Therefore, the transferability analysis indicates that our EST-SSR markers are also potentially useful for correlational research in closely-related species of the genus *Pleurotus*, especially species without sequenced genomes or molecular markers such as *P. eryngii* var. *ferulae*. Furthermore, compared to the genomic SSRs, EST-SSRs have a higher transfer rate in related species [47,48]. The likely reason for this difference is that most of the EST-derived SSRs are originated from transcribed regions, and therefore, they are more conserved across a number of related species within the same genus. Therefore, EST-SSR markers are not only a valuable tool for mushroom breeding, but also a better choice for cross-species phylogenetic studies. 

## 4. Conclusions

We developed a new set of EST-SSR markers, using Bailinggu transcriptome data, which can be used for Bailinggu crossbreeding. Our research contributes the following resources and information: (1) 39, 43 and 34 novel EST-SSR markers that successfully identified monokaryons, differentiated two different monokaryon mating types and discriminated F_1_ and F_2_ hybrid offspring, respectively; (2) monokaryons of Bailinggu have a relatively high genetic diversity; (3) novel EST-SSR markers with relatively high transferability rates in monokaryons of *P. eryngii* var. *ferulae* and *P. ostreatus*; and (4) newly-developed EST-SSR markers that can be used to quickly, easily and precisely differentiate monokaryons from parent dikaryons and identify monokaryon mating types and hybrid dikaryotic strains. Consequently, the newly-developed EST-SSR markers could replace conventional methods for crossbreeding. Ultimately, our study has the potential to significantly enhance breeding research in Bailinggu and other closely-related species. 

## Figures and Tables

**Table 1:** Information about the five strains of *Pleurotus tuoliensis* used for isolating protoplast-derived monokaryons in this study.

No.	Strain Number	Strain Name	Strain Type	Origin
1	CCMJ814	*P. tuoliensis*	Wild strain	Xinjiang, China
2	CCMJ973	*P. tuoliensis*	Wild strain	Xinjiang, China
3	CCMJ980	*P. tuoliensis*	Wild strain	Xinjiang, China
4	CCMJ1077	*P. tuoliensis*	Cultivated strain	Xinjiang, China
5	CCMJ1123	*P. tuoliensis*	Cultivated strain	Shandong, China

**Table 2:** Information about the nine *Pleurotus* strains used for isolating basidiospore-derived monokaryons in this study.

No.	Strain Number	Strain Name	Strain Type	Origin
1	CCMJ967	*P. tuoliensis*	Wild strain	Xinjiang, China
2	CCMJ968	*P. tuoliensis*	Wild strain	Xinjiang, China
3	CCMJ973	*P. tuoliensis*	Wild strain	Xinjiang, China
4	CCMJ974	*P. tuoliensis*	Wild strain	Xinjiang, China
5	CCMJ1001	*P. tuoliensis*	Wild strain	Xinjiang, China
6	CCMJ1002	*P. tuoliensis*	Wild strain	Xinjiang, China
7	CCMJ1044	*P. tuoliensis*	Cultivated strain	Beijing, China
8	CCMJ970	*P. eryngii* var*. ferulae*	Wild strain	Xinjiang, China
9	CCMJ1080	*P. ostreatus*	Cultivated strain	Changchun, China

**Table 3:** General summary of Bailinggu transcriptome sequencing.

Items	Number
Total raw data	199.81 million
Total clean data	177.98 million
Total number of assembled unigenes	31,139
Total size of examined sequences (bp)	60,265,141
Total number of identified SSRs	1110
Number of sequences containing SSRs	2211
Number of sequences containing more than one SSR	165

SSR: simple sequence repeat.

**Table 4:** Characteristics of 18 polymorphic SSR markers used for the phylogenetic analysis of the 37 monokaryons of Bailinggu.

Locus	Motif	Primer Sequence (5′-3′)	Na	Ne	PIC	I
*blg*SSR11	(CCAGGA)11	F-CTTGAATATTGACGGGAGCC R-AGGCTGTGGATAAGAACCCC	4	2.603	0.555	1.011
*blg*SSR13	(CCA)5(CAC)6(CCT)6	F-TCCATAATTGTCATCTCCCCA R-AAATAATTACGACGGTGGGC	5	3.802	0.719	1.457
*blg*SSR14	(AT)7	F-GTCAAACTGCCCAAATTCGT R-CACGGTTGCTCGATTATTCTG	6	2.901	0.612	1.034
*blg*SSR16	(AC)7	F-ACTGCGTCGTGGTGTACAAG R-GTCGCTAAGTATCGGTGGGA	4	2.042	0.607	0.616
*blg*SSR20	(AG)7	F-CGGTGCCAGTGTTCCTTATT R-TGATTCCTGCCCTTTCATTC	5	2.344	0.413	0.708
*blg*SSR24	(GA)7	F-ATCGGGAATGGCAATCAATA R-CTCGAGTCCCGAAACCAATA	7	4.872	0.766	1.586
*blg*SSR26	(TCC)6	F-CCCCGTCCTCTAGTTCATCA R-AATACGGGTCGTCAGATTCG	5	3.044	0.682	1.166
*blg*SSR27	(GGA)8	F-ATCGAAAAATGATTACGCCG R-GGCGAATTTCCTCTTTAGGG	4	2.201	0.602	0.765
*blg*SSR28	(GCG)6	F-ACTTTCCAACACCAACTGCC R-GACCACGAGAGTATCGGAGC	4	3.276	0.650	1.208
*blg*SSR29	(GAG)7	F-TGTTGCAAGCAATGGAATGT R-GTGAATTCCAGCGGTTGTTT	5	3.293	0.646	1.247
*blg*SSR30	(ACC)6	F-GAAGAGTCGCAGACCTCCAC R-GATGCTTTGGTGGACTTGGT	3	1.995	0.431	0.83
*blg*SSR31	(CGC)5	F-AACATGTTCTCCAAGGCCAC R-ATGCGTGCTAACTGAACGTG	4	2.222	0.494	0.844
*blg*SSR32	(ATC)6	F-GCGGATAACCTACTCGTGGA R-ACGCCGAAAATTTTGATGTC	5	3.006	0.612	1.184
*blg*SSR37	(CT)6	F-GAGCCCAAGTGACGTTCCTA R-CTAGGTGGGACTCCGAAACC	5	2.377	0.603	0.993
*blg*SSR38	(AGC)6	F-ATAACAAGGCATGTTTCCGC R-ACAGTCAGGCTCTGGGAGAA	5	3.551	0.690	1.309
*blg*SSR43	(TCC)5	F-CTCAGCCCCAAATTGAACAT R-CTAGTGGCGGGAAGACTGAG	4	2.333	0.584	1.035
*blg*SSR56	(CCA)6	F-GTGTGGGCAGTCGTAGCATA R-CCCTTCCGTTGCTTTCATTA	6	20987	0.525	1.024
*blg*SSR57	(TCC)6	F-CTCGACTCCACGAAAGAAGG R-AACACCCGAAATACGAATGC	4	1.734	0.321	0.635
Mean			4.778	2.805	0.584	1.036

Na: observed number of alleles per locus; Ne: effective number of alleles per locus; PIC: polymorphic information content; I: Shannon’s information index.

## Supplementary Materials

The following are available online at www.mdpi.com/2073-4425/8/11/325/s1. Table S1: Characteristics of 100 simple sequence repeat (SSR) markers of Bailinggu used in this study. Table S2: Information for the 37 monokaryons used in the genetic diversity analysis. Figure S1: The natural genetic recombination found in the dikaryotic strains of Bailinggu, based on the PCR amplification products using four EST-SSR primers and non-denaturing PAGE.
