# Supplementary material for: Development of Novel Polymorphic EST-SSR Markers in Bailinggu (Pleurotus tuoliensis) for Crossbreeding
Source: Genes (Basel). 2017 Nov 17;8(11):325. doi: 10.3390/genes8110325 (PMC5704238; doi:10.3390/genes8110325)
Supplement: Supplementary file 1 [file genes-08-00325-s001.zip › Figure S1.docx]

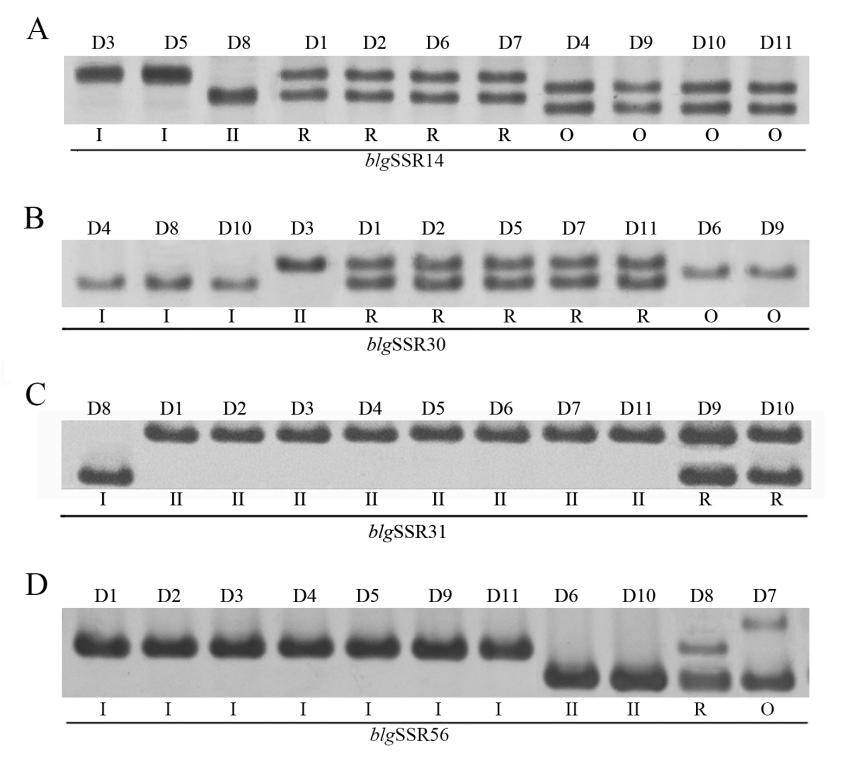


Figure S1. The natural genetic recombination found in the dikaryotic strains of Bailinggu, based on the PCR amplification products using four EST-SSR primers and non-denaturing PAGE. A-D represent the PCR amplification products of four EST-SSR primers including *blg*SSR14, *blg*SSR30, *blg*SSR31, and *blg*SSR56, respectively. I: genotype I; II: genotype II; R: genetic recombination (R= I + II); O: other genotypes. D1–11 are the dikaryotic strains of Bailinggu: D1-CCMJ814, D2-CCMJ1077, D3-CCMJ967, D4-CCMJ974, D5-CCMJ968, D6-CCMJ1044, D7-CCMJ980, D8-CCMJ1001, D9-CCMJ1002, D10-CCMJ973, and D11-CCMJ1123.
