# Supplementary figures and images for: Development of Novel Polymorphic EST-SSR Markers in Bailinggu (Pleurotus tuoliensis) for Crossbreeding
Source: Genes (Basel). 2017 Nov 17;8(11):325. doi: 10.3390/genes8110325 (PMC5704238; doi:10.3390/genes8110325)

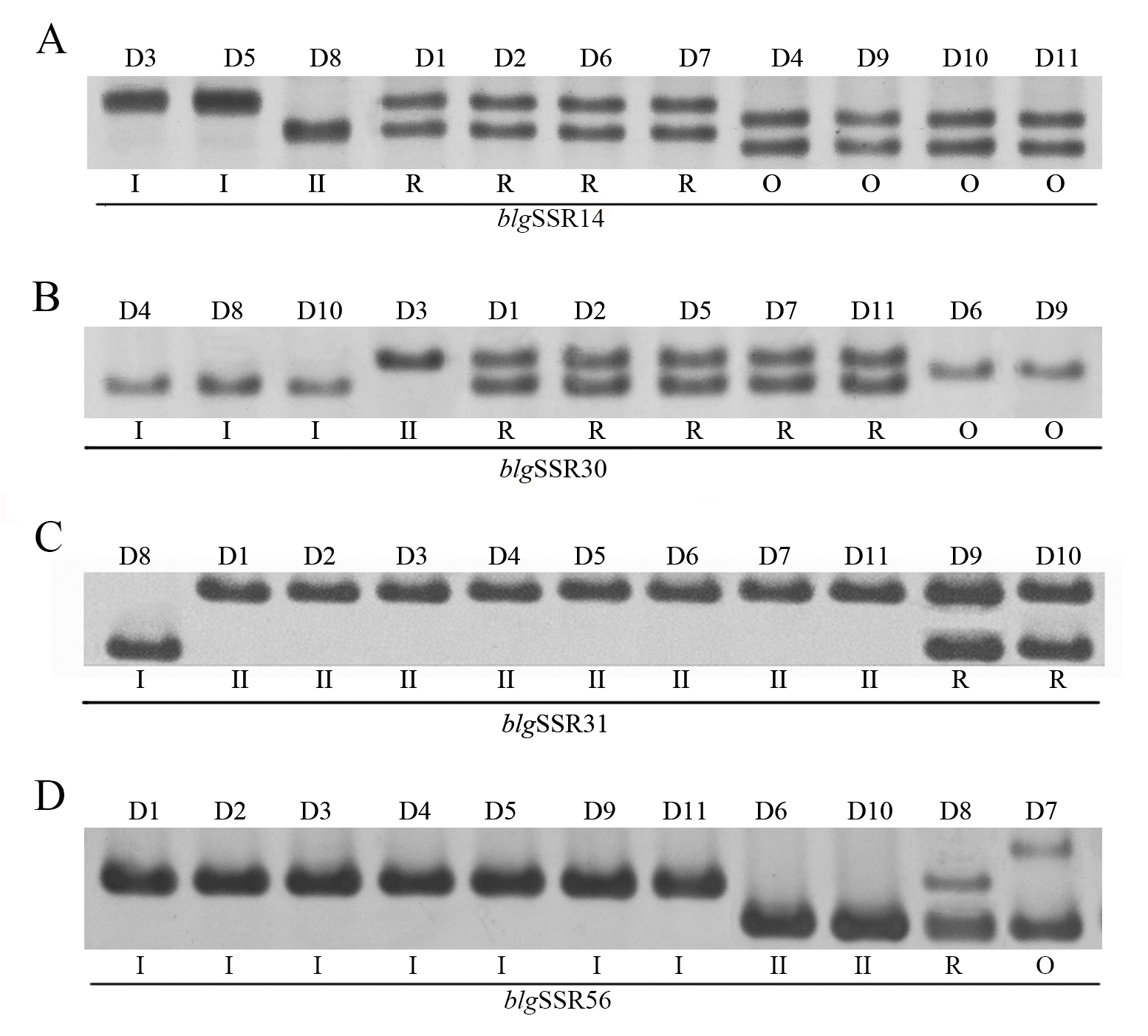

Supplement: Supplementary file 1 [file genes-08-00325-s001.zip › Figure S1.jpg]
